# Diverse Arrangement of Photosynthetic Gene Clusters in Aerobic Anoxygenic Phototrophic Bacteria

**DOI:** 10.1371/journal.pone.0025050

**Published:** 2011-09-20

**Authors:** Qiang Zheng, Rui Zhang, Michal Koblížek, Ekaterina N. Boldareva, Vladimir Yurkov, Shi Yan, Nianzhi Jiao

**Affiliations:** 1 State Key Laboratory of Marine Environmental Science, Xiamen University, Xiamen, China; 2 Institute of Microbiology CAS, Opatovický mlýn, Třeboň, Czech Republic; 3 Department of Microbiology, University of Manitoba, Winnipeg, Canada; 4 Department of Molecular Ecology, Max Planck Institute for Marine Microbiology, Bremen, Germany; Charité-University Medicine Berlin, Germany

## Abstract

**Background:**

Aerobic anoxygenic photototrophic (AAP) bacteria represent an important group of marine microorganisms inhabiting the euphotic zone of the ocean. They harvest light using bacteriochlorophyll (BChl) *a* and are thought to be important players in carbon cycling in the ocean.

**Methodology/Principal Findings:**

Aerobic anoxygenic phototrophic (AAP) bacteria represent an important part of marine microbial communities. Their photosynthetic apparatus is encoded by a number of genes organized in a so-called photosynthetic gene cluster (PGC). In this study, the organization of PGCs was analyzed in ten AAP species belonging to the orders *Rhodobacterales*, *Sphingomonadales* and the NOR5/OM60 clade. *Sphingomonadales* contained comparatively smaller PGCs with an approximately size of 39 kb whereas the average size of PGCs in *Rhodobacterales* and NOR5/OM60 clade was about 45 kb. The distribution of four arrangements, based on the permutation and combination of the two conserved regions *bchFNBHLM-LhaA-puhABC* and *crtF-bchCXYZ*, does not correspond to the phylogenetic affiliation of individual AAP bacterial species. While PGCs of all analyzed species contained the same set of genes for bacteriochlorophyll synthesis and assembly of photosynthetic centers, they differed largely in the carotenoid biosynthetic genes. Spheroidenone, spirilloxanthin, and zeaxanthin biosynthetic pathways were found in each clade respectively. All of the carotenoid biosynthetic genes were found in the PGCs of *Rhodobacterales*, however *Sphingomonadales* and NOR5/OM60 strains contained some of the carotenoid biosynthetic pathway genes outside of the PGC.

**Conclusions/Significance:**

Our investigations shed light on the evolution and functional implications in PGCs of marine aerobic anoxygenic phototrophs, and support the notion that AAP are a heterogenous physiological group phylogenetically scattered among *Proteobacteria*.

## Introduction

Aerobic anoxygenic photototrophic (AAP) bacteria represent an important group of marine microorganisms inhabiting the euphotic zone of the ocean. They harvest light using bacteriochlorophyll (BChl) *a* and various carotenoids serving as auxiliary pigments. These phototrophic microorganisms are thought to be important players in oceanic carbon cycling [1]–[3]. Culture-independent studies have shown that marine AAP bacterial communities are mostly represented by *Alpha-* and *Gammaproteobacteria* [4], [5]. Most cultured marine *Alphaproteobacterial* AAPs belong to *Roseobacter* clade and the order *Sphingomonadales*, which includes members of the genera *Erythrobacter* and *Citromicrobium* [6]–[8]. AAP bacterial isolates related to *Gammaproteobacteria* belong to the clade NOR5/OM60 which contains *Congregibacter litoralis* KT71 [9], [10] and strain HTCC2080 [11].

Compared to the oxygenic phototrophs, the anoxygenic species contain a relatively simple photosynthetic apparatus, which consists of a reaction center surrounded by one to three types of antenna complexes [12]. Both aerobic and anaerobic anoxygenic phototrophs have most of the photosynthetic genes organized in a so-called photosynthesis gene cluster (PGC) [13]. The PGC contains genes for the photosynthetic reaction center, light harvesting complexes, BChl and carotenoid biosynthesis, as well as some regulatory factors. Despite the fact that the basic set of genes in PGC is conserved, the gene organization of operons in PGC largely varies among different AAP bacterial lineages. Two conserved subclusters, *crt*-*bchCXYZ*-*puf* (about 10 kb) and *bchFNBHLM*-*IhaA*-*puh* (about 12–15 kb) were identified in PGCs of different phototrophic *Proteobacteria* [14]–[16]. The orientation of the genes in each subcluster was the same, although the gene order could vary slightly (e.g. *pufBA* and *pufLM*). Interestingly, the regulatory elements such as the transcriptional regulator *ppsR* gene were conserved as well, suggesting that the operons in the PGCs are co-expressed. The organization of *puf* (photosynthetic unit forming, approximately 3 kb) operon varies among different AAP bacterial species. The presence/absence of *pufC* and *pufQ*, as well as various gene orders of *puf* genes, were observed [5], [15]–[17]. Further investigation indicated that such gene organization is crucial for environmental adaptation [14].

Despite its diversity, complexity and functional importance for AAP bacteria, a detailed investigation of the gene and operon arrangement of PGC has not been performed in their entirety. In this study, we analyzed the structure and arrangement of PGC in the AAP bacterial genomes available to date, with the aim of addressing the frequency of homologous gene recombination as well as the differences in carotenoid gene composition and biosynthetic pathways.

## Results and Discussion

**Figure 1:**
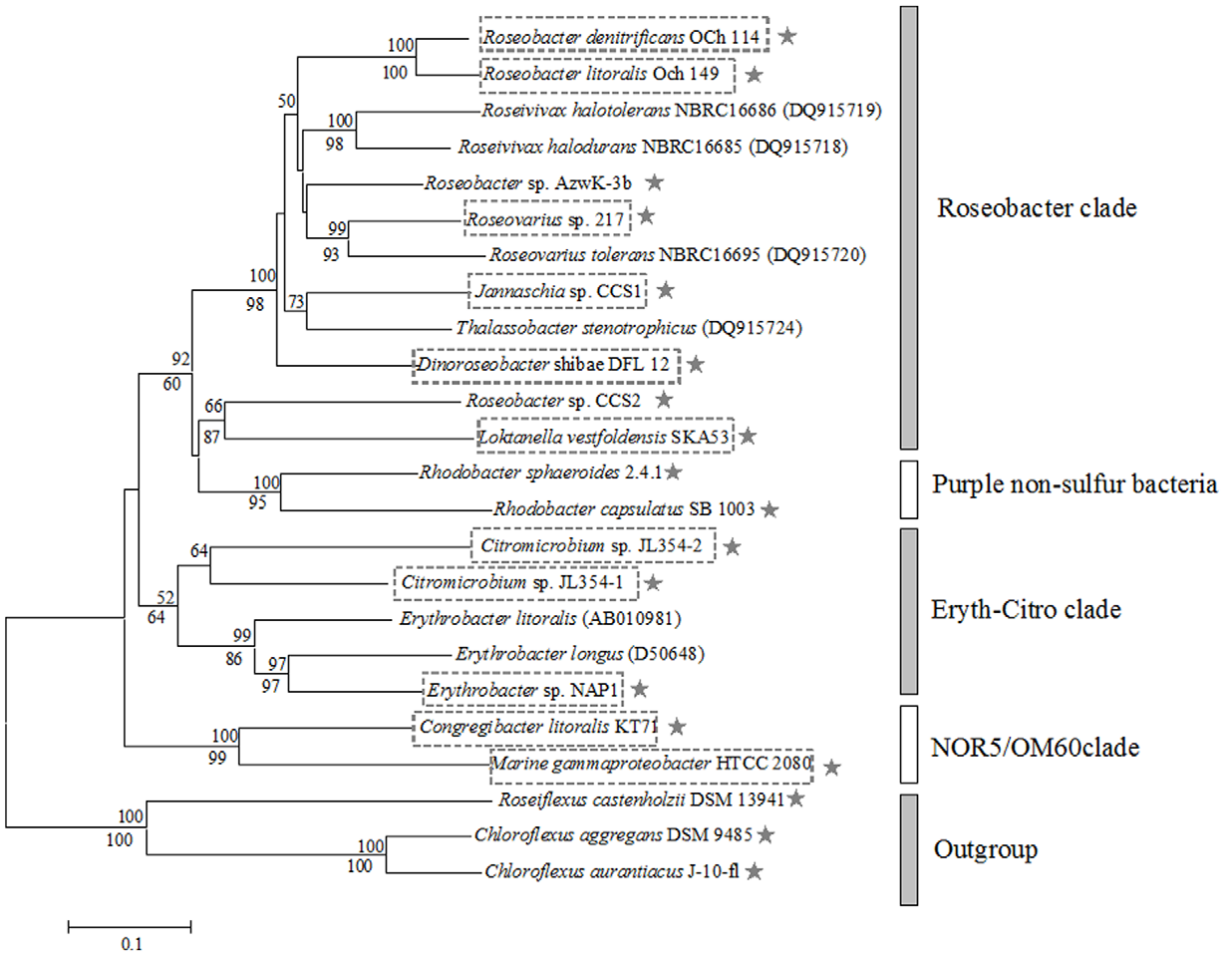
Phylogenetic analysis of *pufM* gene sequences from GenBank database. Symbols “★” represents the *pufM* sequences from whole genome sequence. The whole PGC's of the ten strains highlighted in boxes were also analyzed (Fig. 2). Bootstrap percentages from both neighbor joining (above nodes) and maximum parsimony (below nodes) are shown. Scale bar represents 10% nucleotide substitution percentage.

Ten fully sequenced AAP species were analyzed for their photosynthetic genes and PGC composition. According to phylogenetic analysis using both 16S rRNA and *pufM* genes, the ten strains were classified into three main groups: *Roseobacter* clade (order *Rhodobacterales*), *Erythrobacter*-*Citromicrobium* clade (order *Sphingomonadales*) and NOR5/OM60 clade (*Gammaproteobacteria*) (Fig. 1). *Roseobacter* clade contained six strains belonging to five genera: *Roseobacter (Rsb.) denitrificans* OCh 114 [18] and *Rsb. litoralis* Och 149 [19], *Loktanella vestfoldensis* SKA53, *Dinoroseobacter shibae* DFL12 [20], *Jannaschia* sp. CCS1, and *Roseovarius* sp. 217 [21]. Two species belonged to the order *Shingomonadales*: *Erythrobacter* sp. NAP1 [7] and *Citromicrobium* sp. JL354 [22]. Two species were members of *Gammaproteobacteria*: *Congregibacter (Cb.) litoralis* KT71 [9], [10] and marine *Gammaproteobacterium* HTCC2080 [11]. The genome size varied from approximately 3,064 kb (*L. vestfoldensis*) to 4,763 kb (*Roseovarius* sp. 217). The PGCs represented roughly 1% of the genomes (Table 1). *Sphingomonadales* contained comparatively smaller PGCs (∼39 kb) whereas the average size of PGCs in *Rhodobacterales* and NOR5/OM60 clades was about 45 kb (Table 1). The GC content in the PGCs varied from 52.9% to 66.7%, which was similar to the total GC contents of corresponding genomes (Table 1). This may indicate that PGCs possibly evolve with their genomes long enough to keep homogenous genomic characteristics. The fact that the PGC is a stable part of the phototrophs genome is also indicated also by the fact that the phylogenetic trees constructed for 16S rRNA, *pufM* gene and concatenated PGC core genes show basically the same topology (Fig. 1 and Fig. S2).

**Table 1:** Main characteristics of genomes and PGCs of studied species.

Clade	Organism	Genome size (kb)	PGC size (kb)	Genome GC%	PGC GC%	PGC/Genome
Rhodobacterales	*D. shibae* DFL 12	4,417.8	48.1	65	67	1.09%
	*Rsb. denitrificans* OCh 114	4,331.2	44.6	58	60	1.03%
	*Rsb. litoralis* Och 149	4,678.9	48.3	57	59	1.03%
	*Roseovarius* sp. 217	4,762.6	45.1	60	64	0.95%
	*Jannaschia* sp. CCS1	4,404.0	45.8	62	62	1.04%
	*L. vestfoldensis* SKA53	3,063.7	41.2	59	60	1.34%
Sphingomonadales	*Citromicrobium* sp. JL354	3,273.3	38.7	65	67	1.18%
	*Erythrobacter* sp. NAP1	3,265.3	38.9	61	62	1.19%
NOR5/OM60 clade	*Cb. litoralis* KT71	4,328.1	44.7	58	59	1.03%
	*Gamma*-HTCC2080	3,576.1	43.6	51	53	1.22%

GC% = relative percentage of guanine and cytosine nucleotides. PGC/Genome = PGC as % of genome size.

### The structure and arrangement of PGC

The PGCs have a mosaic structure and consist of five main sets of genes: *bch* genes encoding enzymes of BChl*a* biosynthetic pathways, *puf* operons encoding proteins forming the reaction centers, *puh* operons involved in the RC assembly, *crt* genes responsible for biosynthesis of carotenoids and various regulatory genes. A core set of 27 genes were identified, which were present in all analyzed PGCs (Fig. S1). Most of them came from the BChl *a* biosynthetic pathway. The genes *bchBCDFGHILMNOPXYZ* and *ascF*, with exception of 8-vinyl reductase, represent the complete biosynthetic pathway from protoporphyrin XI to BChl *a*. In contrast, there are only two genes involved in carotenoid synthesis which are common for all PGCs. Other shared core genes encode proteins *pufABLM* and assembly factors *puhABCE* and *lhaA* of the bacterial photosynthetic units.

More complete PGC structures are observed in AAP of *R*oseobacter clade compared to *Sphingomonadales* or NOR5/OM60 clades. The majority of *Roseobacter*-related species contained all the *puf* genes organized in *pufQBALMC* operon, which is involved in the assembly of the photosynthetic units. The only exception was *L. vestfoldensis* SKA53, in which some photosynthetic genes are located outside the PGC and spread throughout the genome. Previously it was reported that the PGC in *Rsb. litoralis* Och 149 is located on a linear plasmid, with two *RPA* genes between *bchFNBHLM-LhaA-puh* and *crtF-bchCXYZ-puf*, which act as a centromere-like anchor when plasmids replicate [19], [23].

The PGC organization in *Erythrobacter* sp. NAP1 and *Citromicrobium* sp. JL354 (order *Sphingomonadales*) is almost identical in terms of gene arrangement and composition. When compared to Roseobacters, this group contains less carotenoid genes and no light-harvesting 2 (LH2) genes. The presence of a smaller number of photosynthetic genes in *Sphingomonadales* is consistent with the smaller size of their PGCs (Table 1).

Similarly, PGCs of two NOR5/OM60 strains have very comparable gene composition and organization. It contains less transcriptional regulators compared to the other groups. Conversely, a BLUF (blue light using flavin adenine dinucleotide sensors) was usually observed in upstream regions of PGCs of NOR5/OM60 clade [9].

**Figure 2:**
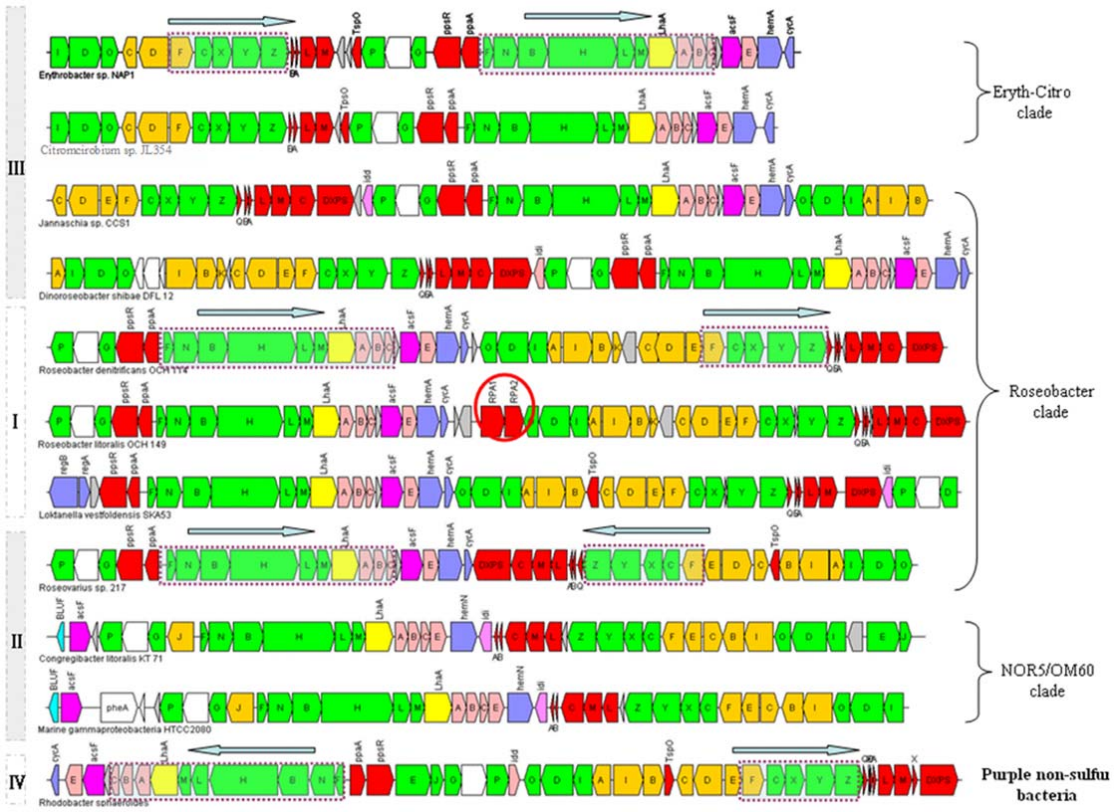
Photosynthetic gene cluster structure and arrangement in AAP species. Green, *bch* genes; red, *puf* and regulators genes; pink, *puh* genes; orange, *crt* genes; blue, *hem* and *cyc* gene; yellow, *LhaA* gene; blank, uncertain or unrelated genes; grey, hypothetical protein. The horizontal arrows represent putative transcripts.

Two conserved gene arrangements are found in all analyzed PGCs: *bchFNBHLM-LhaA-puhABC* and *crtF-bchCXYZ* (Fig. 2). According to their direction and order, the ten PGCs can be divided into three groups: Type I (forward *bchFNBHLM-LhaA-puh* plus forward *crtF-bchCXYZ-puf*) includes *Rsb. denitrificans* OCh 114 and *Rsb. litoralis* Och 149. *Cb. litoralis* KT71, *Gammaproteobacterium* HTCC2080 and *Roseovarius* sp. 217 belong to type II (forward *bchFNBHLM-LhaA-puh* plus reverse *crtF-bchCXYZ-puf*), and the last five organisms form type III (forward *crtF-bchCXYZ-puf* plus forward *bchFNBHLM-LhaA-puh*). The last possible arrangement (type IV, reverse *bchFNBHLM-LhaA-puh* plus forward *crtF-bchCXYZ-puf*) has not been yet found in AAP (or AAP candidates) bacterial genomes (Fig. S1), however it is present in the purple non-sulfur anaerobic bacteria *Rba. sphaeroides* and *Rba. capsulatus* (Fig. 2 and Fig. S2). The distribution of PGC types does not correspond to their phylogenetic affiliation. For example, the *Roseobacter* clade shows all three PGC arrangement types observed in AAP genomes. This suggests that complex operon recombination in PGC occurred after phylogenetic divergence of AAP bacterial genera.

There are four conserved regions in PGCs for BChl *a* expressing of AAP bacteria: *bchFNBHLM*, *bchCXYZ*, *bchIDO* and *bchOP*. Gene *bchEJ*, which exists in most *Rhodobacter*, was found in *Cb. litoralis* KT71 (Fig. 2). There are carotenoid genes between *bchCXYZ* and *bchIDO*, except in *D. shibae* DLF 12 and *Jannaschia* sp. CCS1. The region between *bchOP* and *bchFNBHLM* is of variable sequences in different AAP bacteria clades. In *Roseobacter* clade and *Sphingomonadales*, there are two regulators (*ppsR* and *ppaA*) which are sensitive to light intensity and oxygen concentration [24]. In NOR5/OM60 clade, a *crtJ* gene was found, which controls aerobic repression of BChl, carotenoid, and LH2 gene expression [25], [26].

Four structural types of *puf* gene organization were observed in the ten PGCs: *pufQBALMC*, *pufQBALM*, *pufBALM* and *pufLMCBA*. Unlike the purple non-sulfur species *Rba. sphaeroides* and *Rba. capsulatus*, all the AAP strains studied lack the *pufX* gene in the PGC. The *pufQ* gene, is absent in the *puf* operon of NOR5/OM60 and *Sphingomonadales* clades. In addition, *Sphingomonadales* and *L. vestfoldensis* SKA53 do not have a *pufC* gene. The gene encoding 1-deoxy-D-xylulose-5-phosphate synthase (DXPS) is always located downstream of *puf* genes in the *Roseobacter* clade. DXPS is part of a mevalonate-independent pathway for isopentenylpyrophosphate (iPP) biosynthesis, a precursor for carotenoid and bacteriochlorophyll biosynthesis [27]. Interestingly, a switch of order in the *puf* gene cluster is observed in NOR5/OM60 clade (*pufLMC-BA*) compared to the other two AAP clades (*pufBA-LMC*).

The structure of *puhABC-hyp-ascF-puhE* is conserved in *Roseobacter* and *Sphingomonadales* clades. However in NOR5/OM60 clade, *puhABC* and *puhE* are located together and *ascF* is at a site near BLUF. LhaA, encoding a possible LHI assembly protein [28], occupies the upstream region of *puh* genes. In downstream *puh* genes, there are *hemN* (NOR5/OM60 clade) or *hemA* (*Roseobacter* and *Sphingomonadales* clade) [29], [30].

### The composition and organization of carotenoid genes in PGC

The main difference among analyzed PGCs was found in the genes encoding the carotenoid biosynthetic pathway. The standard set of *crt* genes identified in *Rba. capsulatus* contains *crtAIBKCDEFJ* (Table 2). A slightly reduced set of genes (*crtAIBCDEF*) was also found in some *Roseobacter* species (Table 2). However, the organization of the *crt* operon in *Roseobacter* clade is most variable among PGCs (Fig. 2). The almost complete structure *crtAIBK-hyp-crtCDEF* is present in the genera *Roseobacter* and *Dinoroseobacter* (Fig. 2), while in *D. shibae*, *crtA* and *crtIBK* are separated. Homologous recombination occurred between *crtAIB* and *crtCDEF* in *Jannaschia* sp. CCS1. Comparably, *crtICDEF* and *crtCDF* are missing in NOR5/OM60 clade and order *Sphingomonadales*, respectively. The re-arrangement of *crt* genes may result from events of gene duplication and loss, accounting for the absence of *crtA* gene in *Sphingomonadales* and NOR5 clade (Table 2), and duplication of some of the *crt* genes, such as the *crtE* and *crtIB* found outside the PGC in *Bradyrhizobium* sp. ORS278 [31].

**Table 2:** The composition of carotenoid genes in AAP bacteria.

	*Alpha*				*Gamma*
	*Rhodobacterales*		*Sphingomonadales*		NOR5
	1	2	3	4	5
*crtA*	**•**	**•**			
*crtI*	**•**	**•**	**○**	**○**	**•**
*crtB*	**•**	**•**	**○**	**○**	**•**
*crtC*	**•**	**•**	**•**		**•**
*crtD*	**•**	**•**	**•**		**○**
*crtE*	**•**	**•**	**○**	**○**	**•**
*crtF*	**•**	**•**	**•**		**•**
*crtJ*		**•**			**•**
*crtY*			**○**	**○**	
*crtZ*			**○**	**○**	
*crtW*				**○**	

1, *Roseobacter* clade (*R. denitrificans* Och114, *R. litoralis* OCh149, *D. shibae* DLF 12, *L. vestfoldensis* SKA53, *Jannaschia* sp. CCS1, *Roseovarius* sp. 217 included). 2, *Rhodobacter* genus (*Rhodobacter sphaeroides* 2.4.1 and *Rhodobacter capsulatus* BEC404). The *crtJ* gene was found only in *Rhodobacter capsulatus* BEC404 and NOR5/OM60 gamma-proteobacteria. 3, *Erythrobacter* sp. NAP1. 4, *C. bathyomarinum* JL354. 5, NOR5 clade (*Cb. litoralis* KT71 and *Gamma-proteobacterium* HTCC2080 included). The genes located in the PGC are marked by “**•**”; the genes outside PGC are marked by “**○**”. The gene *CrtK* is not included in the Table as it does not participate in any known carotenoid biosynthetic pathway.

### The biosynthetic pathway for carotenoids in AAP bacterial strains

**Figure 3:**
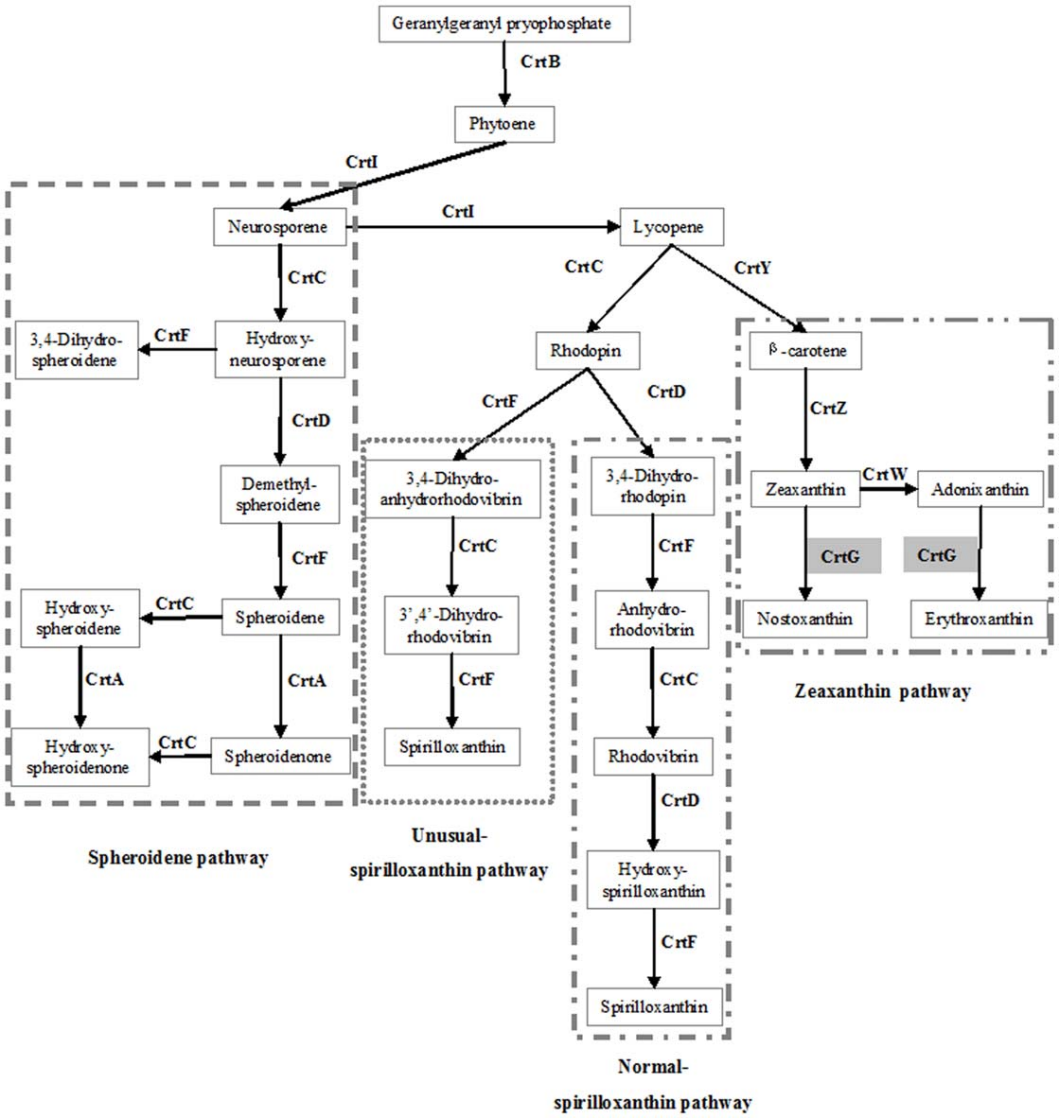
Tentative carotenoid biosynthesis in AAP bacteria. The biosynthetic pathways are proposed based on the identification of respective genes in the analyzed genomes.

A typical feature of AAP bacteria is their pigmentation due to abundant carotenoids, which spans from yellow/orange to brown or from pink/red to purple. While some of the carotenoids serve as harvesting pigments, most of them do not participate in the light harvesting likely having a photoprotection function [32], [33]. As suggested earlier, spheroidenone is the main light harvesting carotenoid in Roseobacters [34]–[36] (Table S1). Spheroidenone is also produced by anaerobic purple non-sulfur photoautotrophic organisms such as *Rba. sphaeroides* or *Rhodovulum. marinum* when grown under aerobic conditions [37], [38]. This is consistent with the closer phylogenetic relationship of these two organisms to Roseobacter related photoheterotrophic species (Fig. 1 and Fig. S2). This indicates the presence of the same carotenoid biosynthetic pathway in all *Rhodobacterales*. The central biosynthetic pathway for carotenoids in the *Roseobacter* clade is the spheroidene pathway (Fig. 3, Table S1), and all the necessary genes (*crtAIBCDF*) for it are located in the PGCs (Fig. 2 and Table 2).

In most studied *Erythrobacter* species, erythroxanthin sulfate was shown to be the main carotenoid [7], [39] (Table S1), however, it does not participate in the photosynthetic processes [39]. Light is harvested by other pigments such as bacteriorubixanthinal, zeaxanthin and β-carotene [39]. The main carotenoid identified in *Citromicrobium* sp. JL354 was nostoxanthin (Table S1). We assume that both species share similar carotenoid biosynthetic pathways (Fig. 3). First, β-carotene is produced from lycopene by the action of lycopene cyclase (*crtY* gene product). Zeaxanthin is obtained by two step hydroxylation of β-carotene catalyzed by β-carotene hydroxylase (*crtZ* gene product). Interestingly, the key genes (*crtY* and *crtZ*) for zeaxanthin pathway are not organized in the PGCs, but are spread throughout the chromosome (Table 2). Zeaxanthin is then a starting intermediade for synthesis of both nostoxanthin (in genus *Citromicrobium*) and erythroxanthin (in genus *Erythrobacter*) (Fig. 3).

The major carotenoids in *Cb. litoralis* KT71 is spirilloxanthin, the same as in *Rhodospirillum rubrum* DSM 467^T^ [10] (Table S1). There are two possible options for spirilloxanthin biosynthesis: typical-spirilloxanthin biosynthestic pathway and unusual-spirilloxanthin pathway (Fig. 3). Interestingly, the gene *crtD* was found to be out of PGC in *Cb. litoralis* KT71 (Table 2), indicating that *Cb. litoralis* KT71 might use the shorter unusual-spirilloxanthin pathway.

In summary, this study showed that most of the photosynthetic genes in AAP species were organized in the PGC. Two conserved regions *bchFNBHLM-LhaA-puhABC* and *crtF-bchCXYZ*, were identified in all studied PGC. Based on their orientation we can divide the studied strains into four different groups. The composition of *bch*, *puf* and *puh* genes in the analyzed PGCs was relatively similar, and the main difference was found among *crt* genes. Such variability was mainly connected with different carotenoid biosynthetic pathways present in AAP groups: spheroidenone biosynthetic pathway in Roseobacters, zeaxanthin pathway in *Sphingomonadales* and spirilloxanthin pathway in gammaproteobacterial NOR5/OM60 clade. Our investigation shed light on the evolution and functional implications of PGCs of marine aerobic anoxygenic phototrophs.

## Methods

### Photosynthetic superoperon sequences and phylogenetic analysis

Ten full-length PGC sequences and gene locations were obtained from the GenBank genome database. The GenBank accession numbers are: *Citromicrobium* sp. JL354 (ADAE00000000) [22], *Loktanella vestfoldensis* SKA53 (NZ_AAMS00000000), *Dinoroseobacter shibae* DFL 12 (NC_009952) [20], *Roseobacter denitrificans* OCh 114 (NC_008209) [18], *Jannaschia* sp. CCS1 (NC_007802), *Roseobacter litoralis* Och 149 (NZ_ABIG00000000) [19], *Roseovarius* sp. 217 (NZ_AAMV00000000) [21], *Roseobacter* sp. CCS2 (NZ_AAYB00000000), *Roseobacter* sp. AzwK-3b (NZ_ABCR00000000), *Erythrobacter* sp. NAP1 (NZ_AAMW00000000) [7], *Congregibacter litoralis* KT71 (*Cb. litoralis* KT71) (NZ_AAOA00000000) [9], [10], Marine *gammaproteobacterium* HTCC2080 (NZ_AAVV00000000) [11].

For comparison two anaerobic anoxygenic phototroph *Rhodobacter sphaeroides* strain 2.4.1 (NC_007493) and *Rhodobacter capsulatus* SB 1003 (NC_014034) also were included in the analysis. Another three green sulfur bacteria genome information used to outgroup of phylogenetic tree, and their Genbank accession numbers are *Chloroflexus aggregans* DSM 9485 (NC_011831), *Chloroflexus aurantiacus* J-10-fl (NC_010175) and *Roseiflexus castenholzii* DSM 13941 (NC_009767). In some cases the automatic gene annotation was corrected manually.

Nearly complete *pufM* (>900 bps) genes and 27 core proteins in PGCs were used to construct phylogenetic trees [17]. Both *pufM* gene sequences collected from NCBI database were aligned using Clustal X and phylogenetic trees were constructed using the neighbour-joining and maximum-parsimony algorithms of MEGA software 3.0 [40]. The phylogenetic trees were supported by bootstrap for resampling test with 1000 replicates.

### Pigment analyses

Six strains were used for pigment analyses: *Rsb. denitrificans* OCh 114 (DSM 7001), *Rsb. litoralis* Och 149 (DSM 6996) and *Erb. longus* DSM 6997 were purchased from the DSMZ culture collections. *D. shibae* JL1447, *Cmi. bathyomarinum* JL354 and *Erythrobacter* sp. JL475 were isolated and maintained in the laboratory [22]. The strains were grown in Erlenmayer flasks with organic rich medium [8] at room temperature (25°C) using a light-dark cycle. The grown cells were harvested by centrifugation and extracted using 100% methanol (in the case of *Sphingomonadales* species) or 7∶2 (vol∶vol) acetone-methanol (in the case of *Roseobacter* species). The pigment extract and analysis were done by high performance liquid chromatography (HPLC) as described previously [41]. Briefly, the chromatography was performed using the Agilent 1100 Series system (Agilent Technologies Inc., Palo Alto, CA, USA). Pigments were separated on a heated (35°C) Phenomenex Luna 3 µ C8(2) 100 Å column with binary solvent system (0 min 100% A, 20 min 100% B, 25 min 100% B, 27 min 100% A, 30 min 100% A; A: 70% methanol+28 mM ammonium acetate, B: methanol) and detected by a UV-VIS diode-array detector (Agilent DAD 61315B).

## Supporting Information

Figure S1 Photosynthetic gene cluster structure and arrangement in other phototrophs. Green, *bch* genes; red, *puf* and regulator genes; pink, *puh* genes; orange, *crt* genes; blue, *hem* and *cyc* gene; yellow, *LhaA* gene; blank, uncertain or unrelated genes; grey, hypothetical protein.
(PPT)

Figure S2 Neighbor joining phylogenetic analysis of 27 core proteins in PGCs from GenBank database. The core proteins are *bchBCDFGHILMNOPXYZ-crtCF-pufABLM- lhaA-puhABCE-ascF*. Bar, 0.1 substitutions per amino acids position.
(PPT)

Table S1 The major carotenoid composition in AAP bacteria.
(DOC)
