# Supplementary figures and images for: Diverse Arrangement of Photosynthetic Gene Clusters in Aerobic Anoxygenic Phototrophic Bacteria
Source: PLoS One. 2011 Sep 20;6(9):e25050. doi: 10.1371/journal.pone.0025050 (PMC3176799; doi:10.1371/journal.pone.0025050)

## Slide 1
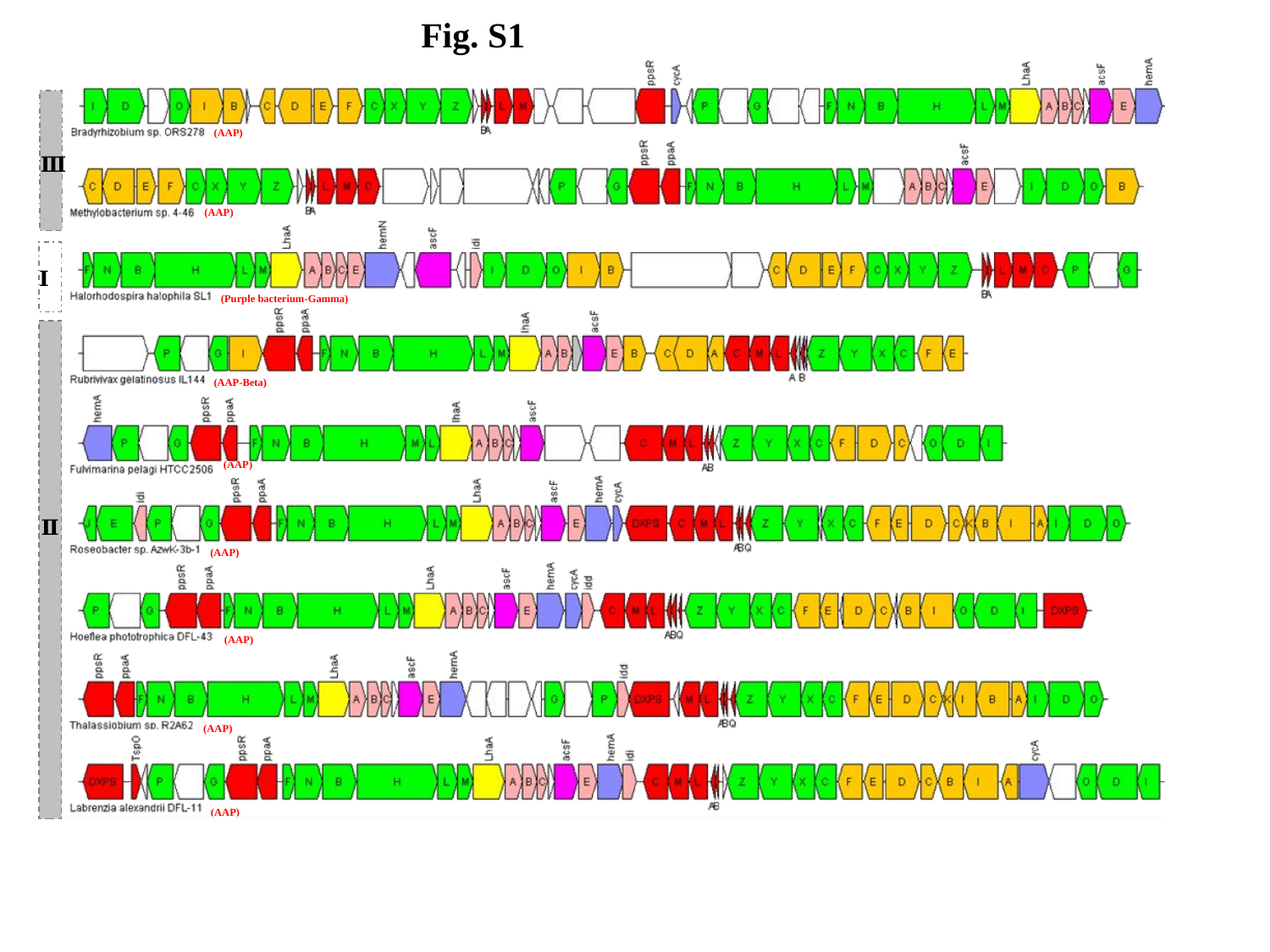

Fig. S1
(AAP)
Ⅲ
(AAP)
Ⅰ
(Purple bacterium-Gamma)
(AAP-Beta)
(AAP)
Ⅱ
(AAP)
(AAP)
(AAP)
(AAP)

## Slide 2
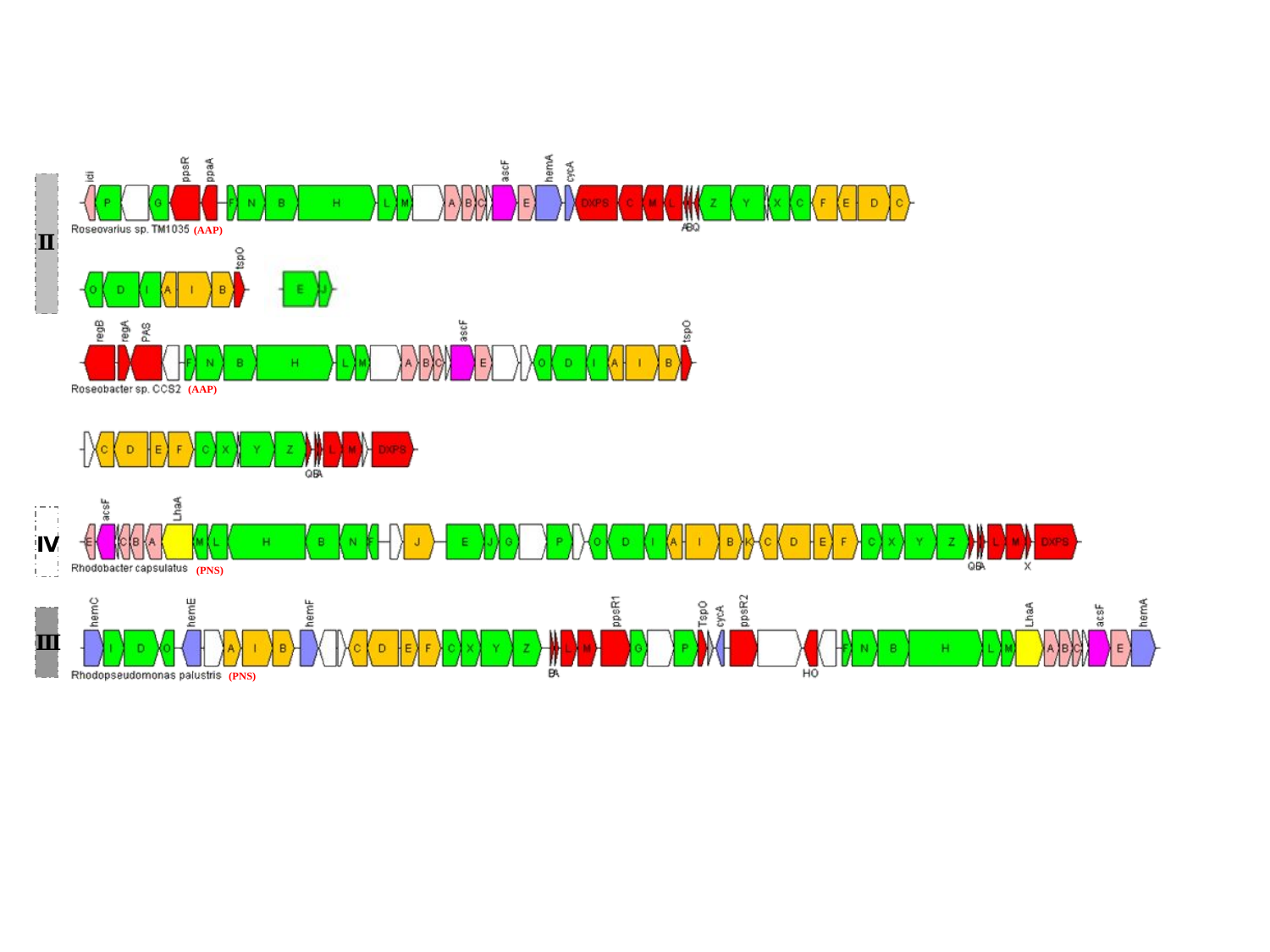

(AAP)
Ⅱ
(AAP)
Ⅳ
(PNS)
Ⅲ
(PNS)

Supplement: Figure S1 — Photosynthetic gene cluster structure and arrangement in other phototrophs. Green, bch genes; red, puf and regulator genes; pink, puh genes; orange, crt genes; blue, hem and cyc gene; yellow, LhaA gene; blank, uncertain or unrelated genes; grey, hypothetical protein. (PPT) [file pone.0025050.s001.ppt]

## Slide 1
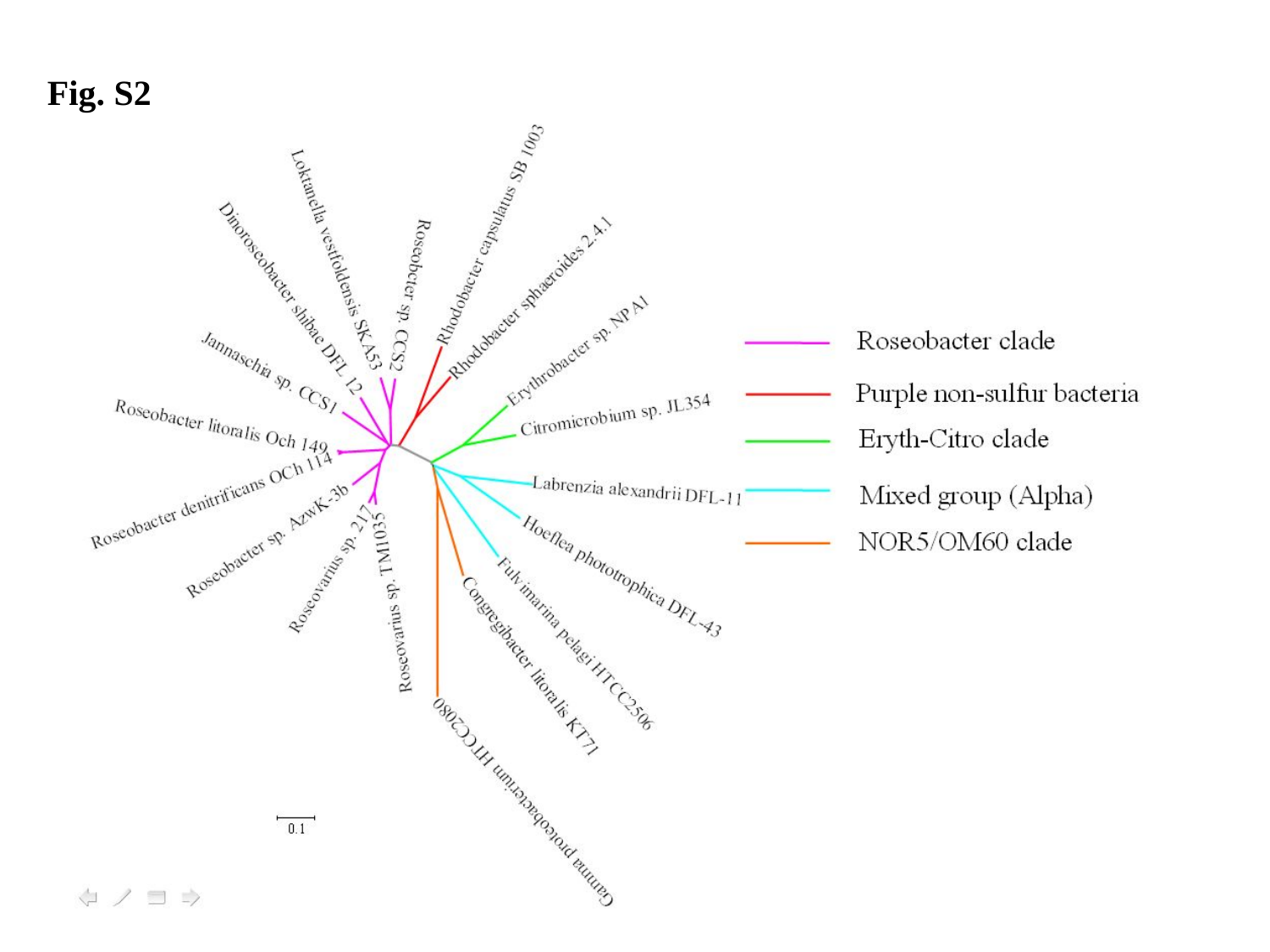

Fig. S2

Supplement: Figure S2 — Neighbor joining phylogenetic analysis of 27 core proteins in PGCs from GenBank database. The core proteins are bchBCDFGHILMNOPXYZ-crtCF-pufABLM- lhaA-puhABCE-ascF. Bar, 0.1 substitutions per amino acids position. (PPT) [file pone.0025050.s002.ppt]
