# Supplementary material for: Diverse Arrangement of Photosynthetic Gene Clusters in Aerobic Anoxygenic Phototrophic Bacteria
Source: PLoS One. 2011 Sep 20;6(9):e25050. doi: 10.1371/journal.pone.0025050 (PMC3176799; doi:10.1371/journal.pone.0025050)
Supplement: Table S1 — The major carotenoid composition in AAP bacteria. (DOC) [file pone.0025050.s003.doc]

**Table S1.** The major carotenoid composition in AAP bacteria.

| AAP bacteria | Strain | Main carotenoids | Reference |
| --- | --- | --- | --- |
| *Roseobacter* clade | *Rsb. denitrificans* Och 114 | Spheroidenone | Takaichi *et al.*, 1991 and this study |
|  | *Rsb. litoralis* OCh 149 | Spheroidenone | This study |
|  | *D. shibae* JL1447 | Spheroidenone | This study |
|  | *Roseobacte*r sp. COL2P | Spheroidenone | Koblížek *et al.*, 2010 |
| *Erythrobacter* | *Erythrobacter* sp. NAP1 | Erythroxanthin sulfate, Bacteriorubixanthinal, Zeaxanthin and β-carotene | Koblížek *et al.*, 2003 |
|  | *Erb. longus* DSM 6997 | Erythroxanthin sulfate, Bacteriorubixanthinal, Zeaxanthin and β-carotene | Noguchi *et al.*, 1992 and this study |
|  | *Erb. litoralis* T4^T^ | Erythroxanthin sulfate, Bacteriorubixanthinal | Yurkov *et al.*, 1994 |
|  | *Erythrobacter* sp. JL475 | Erythroxanthin sulfate, Bacteriorubixanthinal, Zeaxanthin and β-carotene | This study |
| *Citromicrobium* | *Cmi. bathyomarium* JL354 | Nostoxanthin | This study |
| NOR5/OM60 | *Cb. litoralis* KT71 | Spirilloxanthin | Spring *et al.*, 2009 |
